# Retraction Notice to “Anti-tuberculosis drug–induced hepatotoxicity and associated risk factors among patients with pulmonary tuberculosis at a tertiary care hospital in Thailand” [IJID Regions volume 15 (2025) 100665]

**DOI:** 10.1016/j.ijregi.2025.100734

**Published:** 2025-09-15

**Authors:** Sarita Mukura, Parinya Ruenwilai

**Affiliations:** Division of Pulmonary, Critical Care and Allergy, Department of Internal Medicine, Faculty of Medicine, Chiang Mai University, Chiang Mai, Thailand

This article has been retracted: please see Elsevier Policy on Article Withdrawal (https://www.elsevier.com/about/policies/article-withdrawal). This article has been retracted at the request of the Authors and Editor in Chief.

The article is retracted due to issues related to data authorization. The dataset analysed in this study is part of an ongoing collaborative research project and was published prematurely. The authors deeply regret any inconvenience caused to the journal and the broader research community.

